# Reaching the unheard: overcoming challenges in health research with hard-to-reach populations

**DOI:** 10.1186/s12939-024-02145-z

**Published:** 2024-03-18

**Authors:** Venera Bekteshi, Munjireen Sifat, Darla E. Kendzor

**Affiliations:** 1 Dodge Family College of Arts and Sciences, School of Social Work, University of Oklahoma, Norman, OK USA; 2 Sidney Kimmel Cancer Center, Thomas Jefferson University, Philadelphia, PA USA; 3 Department of Family and Preventive Medicine, TSET Health Promotion Research Center, Cancer Prevention and Control Program, University of Oklahoma Health Sciences Center, Stephenson Cancer Center, Oklahoma City, OK USA

**Keywords:** Hard-to-reach populations, Refugees, Immigrants, Health inequities, Cancer screening, Breast cancer

## Abstract

**Purpose:**

Addressing obstacles such as logistical complexities, social stigma, and the impact of historical traumas is essential for the successful inclusion of underrepresented groups in health research.

**Methods:**

This article reviews engagement and interview techniques used to ethically engage recently settled Afghan refugees in Oklahoma and rural Mexican-born women in Illinois in research. The paper concludes with a reflective discussion on the challenges and lessons learned.

**Results:**

Creative strategies to engage hard-to-reach populations in research included considering the participants’ socioeconomic and cultural contexts in their interactions and developing community partnerships to establish trust and obtain reliable data. Other engagement strategies were communicating in the participants’ preferred language, providing assistance with reading and responding to study questions for those with low literacy, employing research staff from the population of interest, and recruiting in specific locations where the populations of interest live.

**Conclusions:**

Community engagement is essential at all stages of research for building trust in hard-to-reach populations, achieving inclusivity in health research, and ensuring that interventions are culturally sensitive and effective.

## Introduction

Hard-to-reach populations such as ethnic and immigrant communities, people experiencing homelessness, refugees, sex workers, and those affected by disasters or conflicts are frequently marginalized in research. This underrepresentation arises from geographic isolation, cultural and linguistic barriers, economic disadvantages, and historical mistrust in healthcare systems of these groups, which translates into a limited or absent understanding of their unique health needs [11, 40]. Yet incorporating diverse communities in research is crucial for a better understanding of the causes of health disparities and achieving health equity. For example, previous diabetes research that included diverse populations, such as African American, Latinx, and Native American communities, led to the development of targeted interventions that factored in the unique dietary, genetic, and socioeconomic characteristics of the affected communities and eventually improved health outcomes within these groups [14]. Similarly, there is evidence to suggest that culturally sensitive mental health interventions for refugees are more accessible and effective when they incorporate preferred languages and consider cultural stigma [30, 37].

However, unlike with populations that have easier access to healthcare systems, engaging with hard-to-reach groups requires a focus on overcoming challenges such as fear of disclosing immigration status, small population sizes, reluctance to discuss health and mental health and medical issues, cultural norms, and mistrust in research [4, 10, 19, 28]. In this context, the purpose of this paper is to share recruitment and assessment strategies, along with the lessons learnt, that we used in our research with hard-to-reach populations—specifically, Afghan refugees and rural Mexican immigrant women living in the United States.

## Literature review

### Recruitment challenges with hard-to-reach populations

Engaging hard-to-reach populations in research poses significant challenges, often resulting in low participation rates and potentially nonrepresentative samples of the target population. Accordingly, researchers working with vulnerable populations, such as immigrants, had to use appropriate sampling frames and culturally sensitive recruitment methods. Recruitment measures with proven effectiveness in facilitating ethical research with hard-to-reach populations include using community-based approaches, employing nonprobability sampling, establishing partnerships with community leaders, and using peer referral systems [1, 13, 26, 34, 36].

Among the effective recruitment strategies used in previous research is involving community leaders of hard-to-reach populations at all stages of research, from conceptualization to intervention development [1]. Here, a particularly beneficial tactic to involving immigrant populations facing accessibility, language, and cultural challenges is Participatory Learning and Action (PLA), which involves establishing partnerships with community leaders in target populations and fostering mutual learning experiences between researchers and the community [25]. Indeed, community partnerships bridge the gap between research teams and immigrants by providing a trusted platform for engagement [14, 18, 25, 31]. Understanding and accommodating participants’ knowledge and experiences renders research more culturally sensitive and relevant, thereby allowing researchers to gain a deeper understanding of the health issues faced by immigrants and other vulnerable populations [25]. For instance, Vahabi et al. [36] employed random sampling in two census tracts with high concentrations of recently transplanted Latin American immigrants. In this study, trusted community members facilitated recruitment in culturally appropriate spaces, such as beauty salons, clinics, grocery stores, and places of worship [36]. Similarly, acknowledging the importance of understanding the cultural traits of immigrants and effectively communicating the benefits of the study to people in the target community, Aglipay et al. [1] recruited community partners who acted as intermediaries and ensured that informative materials were delivered appropriately, thus minimizing potential misunderstandings. The research–community partnership remained strong throughout the research process, leading to successful retention and reliable data. Community partners organized informative workshops to share the results of the study and enhance the project’s impact ([1], see also [36]). This collaborative effort fostered trust, understanding, and relevance, thus amplifying the impact of research on the lives of those being studied [25].

Furthermore, the challenges of low recruitment and high attrition rates among hard-to-reach populations were reported to be effectively handled by using nonprobability sampling methods such as snowball and purposive sampling [1, 19]. In several previous studies, researchers adopted innovative recruitment strategies to enhance the statistical validity of studies, such as respondent-driven sampling (RDS), a recruitment approach to engage individuals from hidden or marginalized populations that combines snowball sampling with network analysis [8, 16]. RDS involves an iterative process that starts with selecting an initial group of participants, known as “seeds”, selected using snowball sampling techniques. Thereafter, “seeds” are asked to refer to other individuals on their social networks to participate in the study. Each new participant recruited through referrals becomes a seed and continues the process, thereby expanding the sample through network connections. RDS leverages social networks to overcome sampling challenges associated with traditionally hard-to-reach populations.

However, in working with immigrant populations, the implementation of random sampling techniques can pose certain challenges, overcoming which requires leveraging alternative approaches. One such alternative approach is the onomastic approach originally proposed by Prandner et al. [28] when recruiting Austrian immigrants by matching their names from specific countries of origin. The onomastic approach involves compiling comprehensive lists of names commonly used in the country of origin of the target population. These lists serve as a reference for selecting individuals who are likely to belong to the desired population. By identifying individuals with names indicative of a particular cultural or national background, researchers can recruit participants with similar characteristics or experiences. However, a limitation of the onomastic approach is that names may be used across multiple cultures; in addition, there are often variations in spelling and pronunciation. To ensure accuracy and reliability of participant selection when employing the onomastic approach, researchers should exercise caution and employ additional verification methods [28].

### Interviewing strategies for hard-to-reach populations

Similarly to difficulties that arise during recruitment, interviewing participants from hard-to-reach populations also poses unique challenges that are distinct from those encountered in more general cohorts. Such challenges include staff capacity limitations, more pronounced due to the need for specialized skills such as language proficiency and cultural competence. Extended efforts in trust-building and navigating complex social dynamics are also essential in this context. Moreover, these challenges are compounded by logistical difficulties of accessing geographically isolated or socially segregated groups. Another critical obstacle is overcoming language barriers that can lead to misunderstandings. In this context, collaboration with advocacy groups and community leaders can be beneficial, as they provide support and insight into the community’s needs [10].

Pilot studies help identify challenges and lead to protocol refinement in response to cultural nuances [19]. Here, an integrated approach is vital to overcome language and communication barriers. Bilingual interviewers, fluent in both the participants’ and researchers’ languages, act as essential mediators, fostering mutual understanding [15, 20]. The roles of translators and cultural brokers are crucial in these efforts. Translators accurately convert languages, while cultural brokers bridge cultural gaps and foster effective communication. Similarly instrumental here are visual aids (e.g., diagrams and pictures), nonverbal cues (e.g., body language), and open-ended questions that encourage participants to express themselves freely and to provide richer and more detailed responses [15].

Finally, establishing safe and supportive spaces for many hard-to-reach research participants requires adopting a trauma-informed approach [10]. By being aware of the potential impact of trauma and prioritizing participants’ emotional well-being and safety, researchers can create an atmosphere that promotes trust and openness. Moreover, recognizing and accommodating diverse communication styles and preferences is integral to effectively unraveling narratives. Each participant may have unique ways of expressing themselves, which requires flexibility and adaptability of part of the interviewer researchers. By integrating these strategies, researchers can create a seamless and effective interview process capable of overcoming barriers to language and communication, respects cultural differences, and facilitates meaningful and inclusive dialogue.

## Case studies

In this section, we outline two research projects we conducted with the following hard-to-reach populations: Mexican immigrant women and newly arrived Afghan refugees. The methods used to recruit and interview participants are discussed, along with the challenges encountered while conducting the research.

### Study 1: contextualizing breast cancer screening among rural and urban Mexican women (Principal Investigator (PI): V. Bekteshi)

The study was approved by the Institutional Review Board (IRB) of the University of Illinois Urbana Champaign prior to initiation, and informed consent was obtained from all participants.

#### Overview

**Fig. 1:**
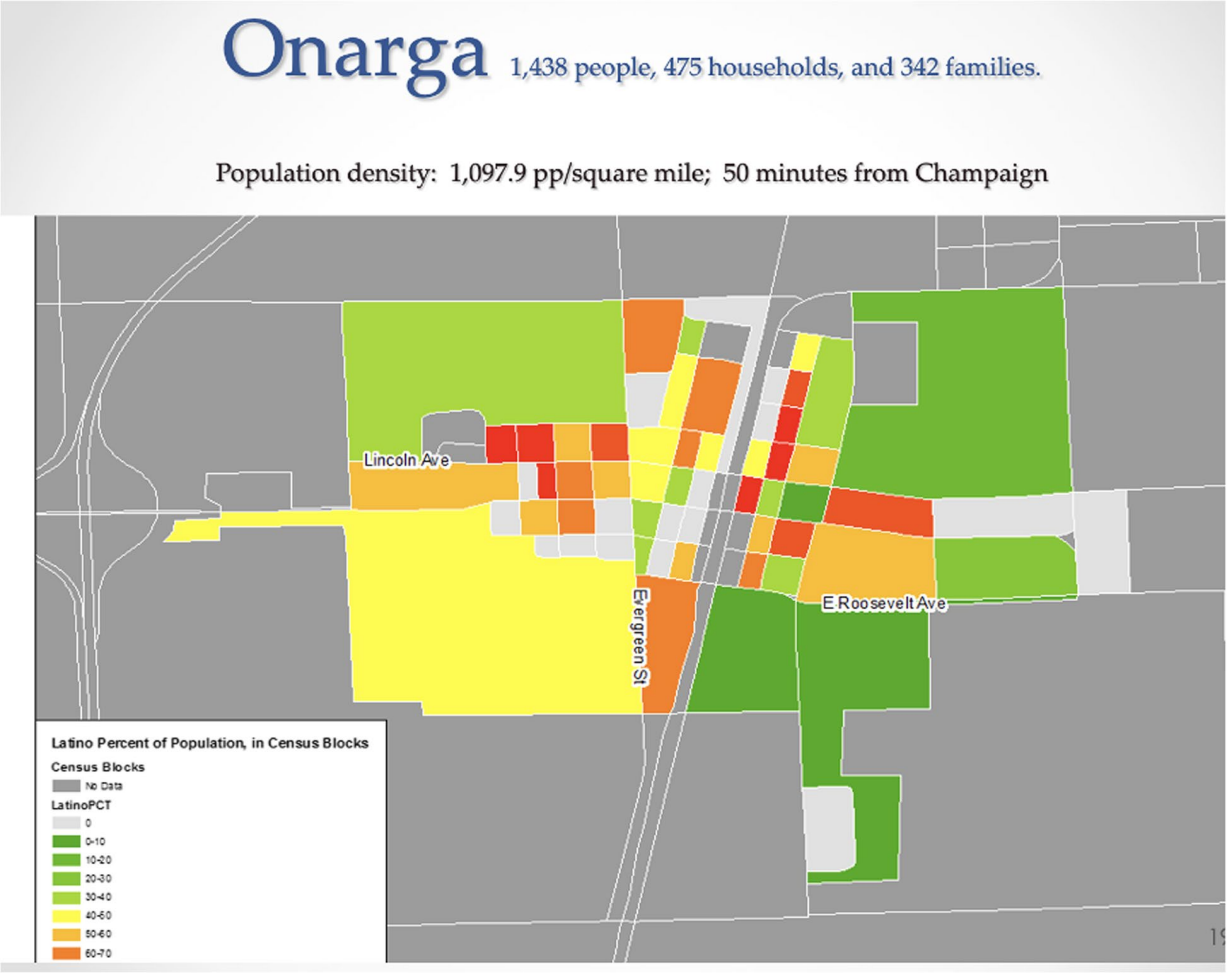
Onarga, IL map: Latino population concentrations in Onarga, IL. The intensity of red color indicates higher numbers of Latinos residing in respective areas

**Fig. 2:**
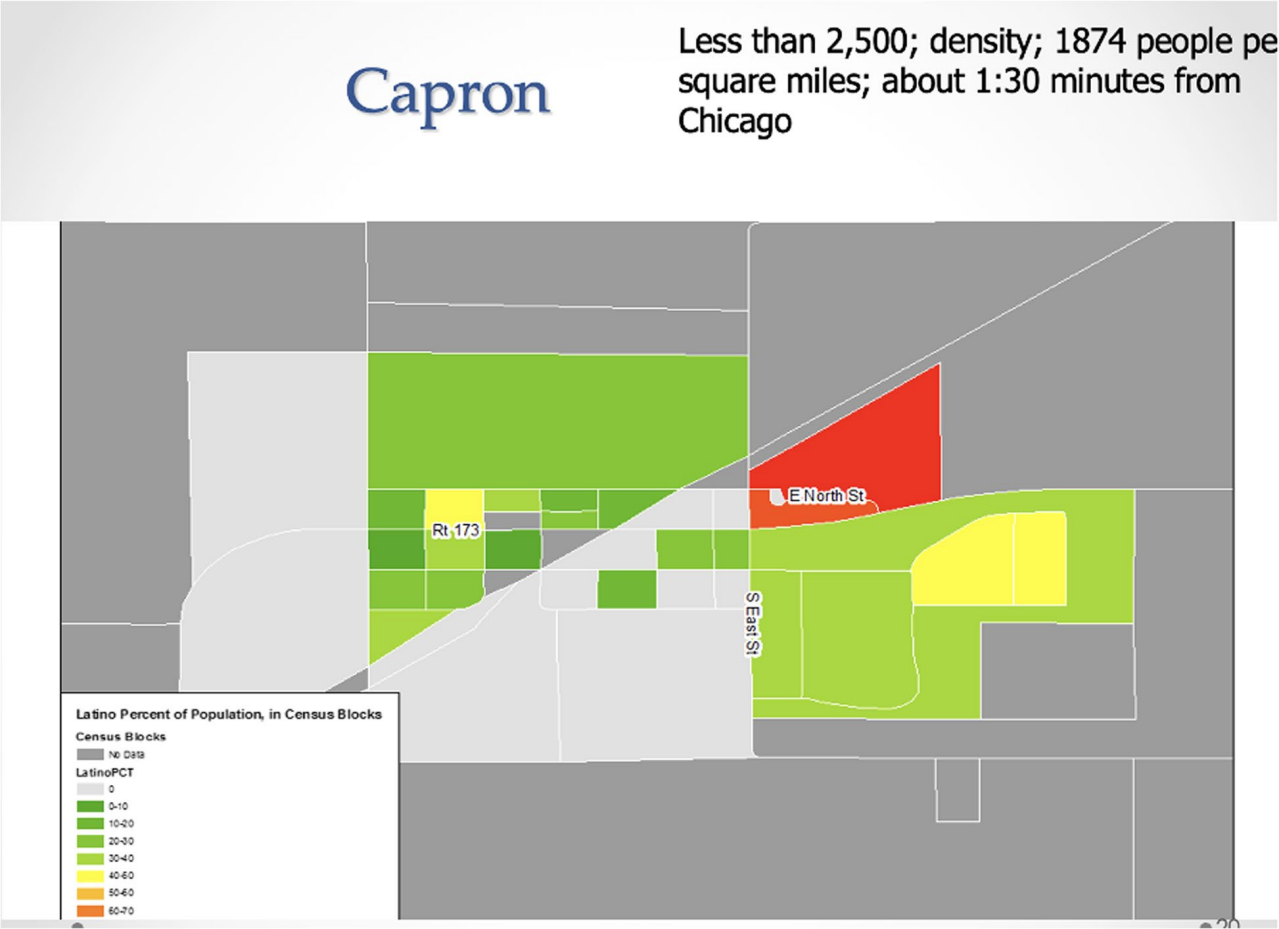
Capron, IL map: Latino population concentrations in Capron, IL. The intensity of the red color indicates higher numbers of Latinos residing in respective area

In the context of the rapidly growing Latinx population in the Unites States, a growing body of research has addressed health disparities affecting Latinos, such as breast cancer screening, access to healthcare, chronic and infectious diseases, maternal and child health, and health behaviors [3, 5, 21, 22, 24, 38]. Some of these disparities arise from structural barriers to healthcare and Latino-specific beliefs [29, 39]. In our first project, we aimed to characterize the influence of Latino-specific beliefs on breast cancer screening within rural communities in Illinois. To this end, we compared traditional cultural beliefs between rural and urban Mexican immigrant women and explored the reasons underlying the lack of engagement in cancer screening among both groups. From April to August 2015, we recruited over 200 Mexican-born women living in rural villages or townships in Illinois, including Onarga (see Fig. 1), Gilman, DePue, Arcola, and Capron (see Fig. 2). Rural areas were defined as geographical entities with a population of fewer than 2,000 inhabitants who were physically distant from central urban areas characterized by a greater access to healthcare services (e.g., Chicago, Ottawa, or Urbana-Champaign, IL). In the remainder of this section, we describe the process of developing the proposal, engaging the community, recruiting Mexican-born women, and interviewing the study participants.

### Pre-engagement phase

#### Literature review

Prior to initiating field work, the PI and the research team conducted a review of the scientific literature to gain a better understanding of the factors affecting the well-being of Latina immigrants, particularly those living in the U.S. This review included insights from the authors’ past work, which shed light on social justice issues faced by Latina immigrants, such as experience of ethnic discrimination and acculturative stress [6]. Moreover, the PI’s extensive experience in working with Latino immigrants [7], along with as her proficiency in Spanish and other languages spoken by the Hispanic immigrant population, predominantly from Mexico, proved to be particularly relevant to the studied population. Furthermore, the PI’s multicultural living experiences, which mirror the backgrounds of the study’s participants, enhanced her ability to engage with and establish trust with them. Likewise, PI’s involvement with the Massachusetts Immigration and Refugee Assistance Coalition (MIRA), a body concerned with policies related to language and social mobility of immigrants, equipped the PI with practical insights and understanding of the community’s unique challenges and needs.

Our team initially focused on building connections with various Latino-serving organizations in the region. Among them, key institutions were the Latino Partnership and La Casa Culture located in Champaign, IL, part of the Urbana-Champaign metropolitan area. Engaging with these organizations was crucial for us to gain a deeper understanding of the cultural contexts surrounding our study participants and to subsequently tailor our recruitment and interview processes to better resonate with the target population. More specifically, this entailed distributing flyers in locations frequented by the Latino community, including houses of worship, Mexican restaurants, and Latino cultural and educational institutions throughout Illinois. Furthermore, we also engaged in consultations with several local organizations known for their work with Latino immigrants, including but not limited to Carle Hospital and Clinic, the Child Care Resource Services Agency, and the East Central Illinois Refugee Mutual Assistance Center, all based in Champaign, IL. The insights gained from these consultations were invaluable in shaping effective engagement techniques.

In addition, we sought the expertise of university faculty members with a background in studying our targeted demographic. Concretely, this involved recruitment of research assistants with direct experience in working within Mexican communities. Accordingly, we enrolled assistants of Mexican ethnicity and a Puerto Rican research coordinator with a history of involvement in projects like the Hispanic Community Health Study/Study of Latinos (HCHS/SOL) [33]. We made this strategic choice in staffing to ensure that our sample would capture the unique experiences and needs of Mexican-born women, rather than generalize across the broader Latina demographic. Such an approach was critical in terms of addressing specific cultural nuances that might impact data collection, such as issues of cultural pride, potential mistrust in researchers, hesitancy in disclosing undocumented residency status, and navigating cultural beliefs like *fatalismo* (the belief in predetermination of life events, including illness) and *harmonia* (a tendency to want to appear agreeable or compliant, which might lead to a bias in responses).

#### Questionnaires

The PI and the research team collaborated with a proficient Mexican Spanish speaker and writer who possessed an in-depth understanding of the educational levels, socio-structural context, and specific cultural traits of Mexican communities in Illinois, as evidenced by her professional experience as a social worker with Latino communities in Illinois. In close cooperation with the writer, we meticulously translated and back-translated the questionnaires to ensure their accuracy and cultural sensitivity. In addition, we conducted a pilot test to verify readability and comprehensiveness of the questionnaire. To this end, a cognitive testing technique [12] was used. The pilot test was conducted with a selected group of 10 Mexican-born women who met our study criteria—namely, age of 40, no cancer diagnosis, and residence in a rural area.

#### Staff training

Graduate research assistants completed the UIUC training focused on human subjects’ protection and a rigorous 16-h training specific to the research project. This comprehensive training covered various crucial topics, particularly obtaining the informed consent form. The researchers were advised to read the consent form at a pace aligned with the participants’ speaking speed, ensuring it was neither too fast nor too slow. Additionally, after every two paragraphs, the researchers were instructed to pause and check in with the participants to inquire about and respond to their questions or concerns. This training emphasized aspects of Mexican culture that might affect the likelihood of individuals’ participation in research and cultivated empathy among the staff towards the participants. We also ensured that graduate research assistants respected autonomy and voluntary participation of the study participants. Specifically, the assistants were instructed to stay attuned to the participants’ facial expressions and promptly address any evident signs of distress. If distress was observed, the staff were instructed to pause the session and inquire whether the participant required a break or had any questions. To better accommodate the participants’ needs, we allowed the participants to be accompanied by their partners during the research process; on top of that, our research assistants were specifically trained to communicate in Spanish. Finally, for the safety of research staff, the presence of a colleague was required during all interviews and survey assessments.

### Engagement phase

#### Recruitment

Initially, we planned to use a random sampling technique, with a particular focus on identifying and inviting participants from specific census tracts prepared by the I.T. Department at the University of Illinois. Overall, this method ensures a broad and unbiased representation of the target population. However, at the stage of implementation of this approach, we faced certain challenges, particularly in reaching and engaging the desired participants within budget and time constraints. These challenges were compounded by a significant increase in the Mexican population in Illinois, leading to changes in community demographics. Specifically, since we were not sufficiently familiar with the neighborhoods, we found that contacting every other household as per the randomization plan was challenging and time-consuming. This lack of local knowledge made it difficult to locate specific households and increased logistical costs.

Recognizing these challenges, we shifted our strategy by engaging a Latino researcher from the University of Illinois in Chicago (UIC) School of Medicine, who had prior experience with similar research projects. This collaboration provided us with valuable insights into Latino culture and access to cultural venues, facilitating a more community-centric approach to recruitment. We also actively advertised the project through churches, non-governmental organizations (NGOs) and clinics serving the Mexican communities in Onarga, DePue, and Capron, IL. In some instances, this collaboration resulted in the implementation of a more direct engagement strategies, such as setting up tables in NGOs and clinics serving the population to personally meet Mexican women, explain them the study, and directly invite them to participate. These face-to-face interactions complemented our broader recruitment efforts and proved effective in engaging the target demographic, as evidenced by the enthusiastic response and participation from these communities, including active participation in initial meetings, a high number of registrations within a short period, and substantial community feedback and engagement in the study’s preparatory activities.

Furthermore, in response to the initial difficulties with random sampling, we shifted to a targeted snowball sampling strategy. This involved conducting interviews with multiple individuals from the same household and consulting with participants to identify nonprofit organizations and places of worship where further potential respondents could be found. We also sought permission from local leaders (e.g., priests, directors) to disseminate information about our study, and workshops were conducted in community-based organizations for further outreach. To better delineate the community boundaries, based on the information from participants and on-site observations, we created a hand-drawn map that reflected the actual locations of Mexican communities in these rural areas. The data collection, carried out from April to August 2016, involved visiting households identified as Latino; yet, our outreach also extended to trailer camps, clinics, and nonprofit organizations, which resulted in recruiting a total of 350 interviewees. This adaptation in recruitment strategy, combined with the expertise of the recruited Latino researcher, enabled us to effectively engage with the Mexican communities in these rural areas and facilitate the continuation of data collection for the Lucha study. One example of our innovative and collaborative effort was the development of custom-designed T-shirts. Created in vibrant green and reminiscent of Mexican soccer jerseys, these T-shirts featured empowering messages along with the “Lucha” logo. This decision emerged from the PI’s close engagement with community member students, reflecting a shared commitment and a powerful symbol of the cause. Symbolizing the collaborative spirit and cultural sensitivity at the heart of our study, these T-shirts strongly resonated with the participants.

#### Interviews

All interviews were conducted in Spanish. We initiated conversations by inquiring about participants’ daily activities and experiences in their new country. Research staff were advised to adopt a relaxed attitude and to adjust their speaking pace to match that of the participants, be it slower or faster. If needed, the researchers were instructed to pause the interview to offer necessary clarification. Whenever the interviewees encountered difficulty in recalling their answers, reassurance and suggested revisiting the questions were provided. During the interviews, we accepted hospitality from participants, like invitations to try Mexican foods and receiving small gifts such as potted plants. These gestures of appreciation were symbolic of the community’s engagement with our study. Upon completion of the project, we not only expressed gratitude by sending thank-you notes to all partners, but also actively shared our findings with the community. To this end, presentations were made in social work classes, local clinics, and specifically in Catholic Churches known for their Spanish-speaking congregations, including St. Mary’s Church in DePue and Our Lady of Guadalupe in Onarga. All these efforts were aimed at ensuring that the study’s results reached both academic circles and the grassroots community, aligning with the project’s commitment to inclusive knowledge dissemination.

### Study 2: understanding the mental health needs of Afghan refugees in Oklahoma: overcoming recruitment challenges and fostering community engagement (Principal Investigator: M. Sifat)

Prior to initiation, the study was approved by the Institutional Review Board (IRB) of the University of Oklahoma Health Science Center. Informed informed consent was obtained from all participants.

#### Overview

In this cross-sectional survey, we examined the prevalence of mental health needs among recently resettled Afghans (*n* = 348) who obtained Special Immigrant Visas enabling them to relocate to Oklahoma upon the withdrawal of U.S. military forces from Afghanistan in August 2021 (Sifat M, Kenney S, Bekteshi V, Kendzor D: Correlates of poor mental health among recently resettled Afghan refugees in Oklahoma city, in preparation). During this research project, we encountered various challenges that, along with the lessons we learned while conducting research in this hard-to-reach population, are outlined below.

#### Security concerns/wariness of strangers

Recruiting Afghan participants for research can be particularly challenging because this demographic is frequently wary of outsiders (see, e.g., [2, 23]). This hesitance stems from Afghan individuals’ unfamiliarity with the research process and concerns about the potential stigma associated with participating in mental health studies [2]. During the study, our participants expressed concerns about sharing their personal information. Many Afghan individuals assisted the U.S. government prior to U.S. withdrawal and were concerned about facing unfavorable treatment by the remaining governing authority in Afghanistan if their association with the U.S. would become known. This situation generated pervasive fear about safety of their families and friends returning home. We managed to overcome this barrier upon reiterating that no personal identifying information would be made public.

#### Challenges for women participating in research

Additional difficulties arose when recruiting female Afghan participants. As indicated in previous research, Afghan women are typically accompanied and guarded by their spouses or family members during interview sessions, which can hinder women’s willingness to participate and their ability to respond freely [23]. In our study, we also observed this pattern. One of our recruitment methods involved offering potential participants the option to schedule assistance from a research assistant for survey administration at a location of their preference, with many choosing the convenience of their homes. However, while removing the need to travel, this facilitation presented a gender-related challenge. In some instances, both husbands and wives were present in the same room during the survey. Therefore, even though the responses remained confidential, the settings where the survey was conducted could have led to bias in the responses. For instance, in one case, a female interviewee hesitated to respond to any survey questions without consulting her husband first. In future research, this barrier may be bridged by hiring a female research assistant for female participants.

### Pre-engagement phase

In this study, we used a consultative approach that involved incorporating feedback from stakeholders and community members prior to data collection. Before the study launch, the PI actively engaged with the Afghan community through volunteer work for nonprofit organizations (The Spero Project). Additionally, unrelated to the study, the PI helped to furnish homes (including collecting and providing home essentials, as well as setting up apartments ready to be moved into) for Afghan individuals relocated to apartment complexes; similarly, the PI also contributed to the development of health-related workshops tailored to the needs of the population (e.g., dental hygiene). Through these interactions with the Afghan community, the target demographic group was already familiar with the PI prior to the start of the project. Owing to these efforts, and in line with previous research that highlighted the importance of establishing trust, accessibility, and rapport within the Afghan refugee community [2, 32], we managed to reach most of the recently resettled Afghan population in Oklahoma City.

Concurrent to these volunteer efforts, the PI also conducted preliminary interviews with various stakeholders, Afghan and non-Afghan alike, including community representatives, healthcare providers, and resettlement personnel. These interviews focused on the following three main areas: (1) gathering information about the demographic composition and size of the Afghan refugee population in the Oklahoma City area; (2) obtaining recommendations on effective strategies for sampling, recruiting, and assessing Afghan individuals’ willingness to participate in a mental health-focused study; and (3) gaining insights into the intricacies of the resettlement process of the target group, including their temporary housing arrangements and access to healthcare and insurance. In these efforts, we followed previous research on Afghans residing in the United States that emphasized the importance of engaging key informants and community gatekeepers through in-depth interviews so as to gain more access to the broader community and increase knowledge of community dynamics [2, 32].

Our interviews with stakeholders provided three valuable insights. First, the primary resettlement agency and the sole nonprofit organization highlighted the need to consider the prospective study participants’ potential low literacy levels and their ability to read and understand the survey questions. Second, the interviewed stakeholder underscored the importance of effectively communicating privacy measures to protect participant data. Thirdly and finally, sensitivity surrounding mental health-related topics and associated stigma were identified as critical factors to be considered.

In parallel to the interviews with stakeholders, the PI developed survey items in English that were then translated by professional translators into Dari and Pashto, two most common Afghan languages. Thereafter, we used the back translation method [9] to ensure translation accuracy. The back translation was performed by a member of the Oklahoma Afghan community, and the outcomes were reviewed by both research assistants to prevent misinterpretations, such as an incorrect translation of “headache” as “swollen head”. The research assistants also identified questions that our target population might consider to be taboo, such as the question regarding sexual activity asked after the question about one’s marital status. The assistants noted that, taking into account the stigma of sexual activity outside of marriage, our prospective participants who were single would not want to answer the questions about sexual activity. Accordingly, the interviewing staff were instructed to skip the question if participants showed any signs of discomfort.

In order to address concerns about low literacy levels of our target demographic, the following two data collection strategies were devised. First, the participants who were literate in English, Pashto, or Dari were given the opportunity to complete the survey independently in their preferred language. Alternatively, a research team member recruited from the Afghan community who was proficient in all three languages read the survey questions and corresponding answer choices to the participants. This process was facilitated by using a smartphone that displayed only one question and its respective answer choices at a time. This approach enabled our participants to privately select their responses before the interviewer proceeded with additional inquiries. To incentivize participation, the participants were offered a $30 department store gift card. Early on in the data collection process, we observed that our participants preferred Walmart gift cards to Target, as their apartment complexes were closer to the former department store Walmart, and many of our respondents did not have personal transportation.

### Engagement-phase

#### Recruitment and interviews

Through active involvement in the community and stakeholders, the PI cultivated a sense of rapport with the target population and identified their preferred socialization areas. Both the nonprofit agency (The Spero Project) and the official resettlement agency (Catholic Charities) extended their support for the research project by providing letters of endorsement for Institutional Review Board (IRB) approval.

We recruited participants following health workshops hosted by the non-profit agency, which were designed specifically for the Afghan population. Subsequently, using a snowball sampling approach, we encouraged the participants to refer to acquaintances, thereby enabling us to arrange further appointments and assist with survey inquiries. The participants interested in participating in the study were offered the opportunity to schedule a convenient survey time at a location of their choice. Many participants opted for research assistants’ coming to their homes, after work hours. Flexibility of our research assistants considerably increased the response rates; furthermore, since many Afghan refugees resettled into the same apartment complexes, information about our study quickly among the target population spread by word of mouth. Of 348 participants recruited, only 10 used self-administration of the survey, while 338 were aided by the research team.

## Discussion

In conducting research with Afghan refugees and Mexican-born women in rural areas, we navigated a series of challenges that reshaped our study methodologies and research perspectives. In adapting to the unique needs of these communities, we considered the critical role of gender dynamics, cultural sensitivity, and ethical research practices. The adaptive measures we took were instrumental in refining interviewing strategies to better match the specific cultural and gender contexts of the participants. A review of these adaptations provided in this paper demonstrates how a nuanced understanding of the study populations’ distinct characteristics can lead to more a more effective engagement and richer insights.

Albeit different in their demographic focus, our two case studies—one targeting Mexican women and the other involving Afghan refugees in Oklahoma—share a common thread of complexities and innovative solutions that demonstrate the intricate nature of conducting inclusive and effective health research. In the research project targeting Mexican women, crucial aspects were the collaboration with community leaders, advocacy groups, and experienced agencies. Serving as both cultural intermediaries and vital conduits, the established partnerships were leveraged to facilitate initial connections, engage potential participants, and efficiently disseminate project information [1, 13, 26, 34, 36]. Our collaborative approach proved instrumental not only in providing dedicated spaces for data collection, but also in creating a culturally sensitive and trustful environment. Through fostering a sense of safety and cultural relevance, this environment was pivotal for meaningful participant involvement, encouraging participants to more openly and meaningfully engage in the research process.

Furthermore, the project with Mexican women faced challenges, particularly in gaining momentum and establishing trust within each data-collection community among Mexican women. Each of the three investigated communities Illinois—Onarga, DePue, and Capron—had distinct environmental characteristics. While DePue featured some densely populated areas, Capron presented challenges in reaching its more dispersed residents. To address these concerns, we sought to establish strong partnerships via engaging community leaders and advocacy groups early on. This proactive involvement provided valuable insights and support, ensuring a collaborative approach tailored to the specific needs of each community [17]. Using culturally sensitive approaches, we also adapted our communication and outreach strategies to effectively engage residents in both densely populated areas like DePue and sparser ones like Capron [35]. The PI and research team invested significant effort into building genuine rapport with participants, demonstrating deep interest in the project and care for the participants’ experiences. By actively listening to and addressing participants’ concerns and respecting their perspectives, we established strong relationships throughout the data collection process [22, 27].

In our project with the recently resettled Afghans in Oklahoma, a key element was our active engagement of community members. Involving community members in the research process led to the development and implementation of culturally sensitive research programs, which provided deeper insight into the studied population [2, 32]. Initiating community engagement early on after the project’s launch, we focused on building rapport and showing genuine interest in the community members’ lives, thereby enhancing the research process and promoting participation in the target population [2, 32].

### Methodological adaptations

In the early stages of community engagement, we carefully reviewed our research activities aimed at establishing trust for potential perceptions of coercion. This concern arose when we observed that our efforts to be acknowledged as trusted community allies inadvertently created pressure on individuals to participate. To address this concern, we refined our research methodology to additionally include a consultative approach, thereby enabling incorporating feedback from community leaders and participants. This adaptation is well aligned with several culturally sensitive and ethical recruitment methods reported in the literature [2, 10].

For instance, in the Lucha Study with Mexican-born women in rural Illinois, we initially used a direct, face-to-face recruitment approach. However, as soon as we realized that this approach might have inadvertently created undue pressure on individuals to participate, we shifted to using an indirect approach, collaborating with local churches and NGOs. This strategy leveraged established community trust and facilitated a culturally respectful and sensitive dissemination of study information, which subsequently improved the recruitment process and deepened our understanding of community dynamics. Similarly, in the study involving Afghan refugees in Oklahoma, the initial research schedule did not align with the schedules of Afghan community stakeholders, who were focused on immediate resettlement tasks. Accordingly, upon discussions with community leaders and resettlement agencies, necessary adjustments were introduced into our research timeline.

### Gender dynamics and interviewing strategies

In both our studies discussed in this paper, gender played a pivotal role in shaping the experiences and health outcomes of the targeted populations. Accordingly, in both recruitment and interviewing techniques, we leveraged a gender-sensitive approach to ensure genuine engagement and accurate data collection [2, 23]. Gender-specific health issues were particularly salient in the context of Mexican-born women in rural areas, where cultural factors significantly influenced breast cancer screening behaviors.

Overall, our studies informed us that traditional beliefs and gender roles were important within the studied communities, which posed unique challenges and required a tailored approach to effectively engage and interview female participants [36]. To address these nuances, we adapted our interviewing techniques. This involved changes to interview settings to ensure comfort and privacy, as well as to respect gender norms and meet our study participants’ expectations. For instance, in the Afghan community, we would frequently conduct our interviews in settings that were deemed culturally appropriate and safe for women. To provide an example, the study participants were not left alone in a room with a male research assistant who they were not related to, and they were able to do interviews from the privacy of their homes.

A reflection on these experiences suggests that future research on hard-to-reach populations requires a careful consideration of gender dynamics and employing appropriate interviewing strategies. Overlooking such gender-related concerns can compromise data reliability. For instance, we observed that some of our female interviewees within the Afghan community were reluctant to engage in a study without spousal consent. Nevertheless, after discussions about data confidentiality, most husbands had no objections to their wives’ participation in the study.

### Reflective practices and community participation

Reflective questioning became an integral part of our research methodology. Before engaging, we critically assessed our assumptions about each of the two studied community. For instance, while we initially assumed the Mexican immigrant women to be comfortable with direct recruitment methods, this approach was revised at a later point. Similarly, we continuously evaluated our methods during the engagement and iteratively adapted our approaches based on community feedback. After the engagement, we also integrated the lessons learned, such as the importance of cultural symbols and group dynamics, into four research practices.

As revealed by a closer look at the two case studies, the nature of community participation varied significantly. Formal partnerships became necessary in situations requiring institutional support and resources. For instance, in the Afghan refugee study, we entered into formal partnerships with resettlement agencies, which granted us access to the studied population; in addition, we also had to align our research goals with their resettlement priorities. Conversely, in contexts where community trust and cultural insights were paramount, as in the Lucha study, informal collaborations proved to be more effective, thus allowing for more organic interaction and trust-building.

Power sharing in decision-making processes was evident in both studies. In the Lucha Study, the PI’s engagement with community member students led to the creation of strategies that resonated with the participants. A result of this collaborative effort was the decision to use T-shirts with empowering messages, which symbolized a shared commitment to the cause. Similarly, a joint effort was the control of resources, as negotiated between our research team and community leaders. In the Afghan study, the survey and recruitment strategy incorporated consultation with community leaders, which was meant to ensure that the used methods were feasible and respectful of the community’s needs. In the Lucha study, resources were allocated for the production of culturally relevant materials, like the T-shirts, which were decided upon in collaboration with community members, ensuring that the benefits of the research were meaningfully and equitably distributed. Overall, our willingness to adapt research design in response to community feedback not only helped us in overcoming logistical challenges, but also enriched our understanding of the cultural dynamics within the Afghan refugee community. These adaptations were implemented as an acknowledgement of the importance of flexibility and cultural sensitivity in conducting research with hard-to-reach populations. The lessons learned from these adjustments are instrumental in guiding further research and emphasize the value of aligning research methodologies with the cultural and practical realities of the communities under study.

## Conclusion

In this paper, we highlighted strategies to engage hard-to-reach populations, with a particular focus on the necessity of community engagement to facilitate responsiveness to cultural sensitivities. Building trust and strong relationships is vital for collaboration. As demonstrated by our two case studies, future studies should prioritize culturally appropriate translations, accommodate cultural norms, and respect individual routines and social structures. Adoption of such approaches can effectively enhance research inclusivity and methodology, thereby contributing to health research and ensuring that the results are representative of and relevant to diverse populations.

## Data Availability

Data are available upon reasonable request with the approval of the University of Oklahoma Institutional Review Board (IRB) by contacting the corresponding author (VB).
